# The Recovery from Sulfur Starvation Is Independent from the mRNA Degradation Initiation Enzyme PARN in Arabidopsis

**DOI:** 10.3390/plants8100380

**Published:** 2019-09-27

**Authors:** Laura Armbruster, Veli Vural Uslu, Markus Wirtz, Rüdiger Hell

**Affiliations:** Centre for Organismal Studies, Heidelberg University, 69120 Heidelberg, Baden-Württemberg, Germany; laura.armbruster@cos.uni-heidelberg.de (L.A.); veli.uslu@cos.uni-heidelberg.de (V.V.U.); markus.wirtz@cos.uni-heidelberg.de (M.W.)

**Keywords:** AGS1, AHG2, sulfur starvation, PARN, recovery, sulfate transporters, sulfate resupply, mRNA degradation, rapid recovery downregulation

## Abstract

When plants are exposed to sulfur limitation, they upregulate the sulfate assimilation pathway at the expense of growth-promoting measures. Upon cessation of the stress, however, protective measures are deactivated, and growth is restored. In accordance with these findings, transcripts of sulfur-deficiency marker genes are rapidly degraded when starved plants are resupplied with sulfur. Yet it remains unclear which enzymes are responsible for the degradation of transcripts during the recovery from starvation. In eukaryotes, mRNA decay is often initiated by the cleavage of poly(A) tails via deadenylases. As mutations in the poly(A) ribonuclease PARN have been linked to altered abiotic stress responses in *Arabidopsis thaliana*, we investigated the role of PARN in the recovery from sulfur starvation. Despite the presence of putative PARN-recruiting AU-rich elements in sulfur-responsive transcripts, sulfur-depleted PARN hypomorphic mutants were able to reset their transcriptome to pre-starvation conditions just as readily as wildtype plants. Currently, the subcellular localization of PARN is disputed, with studies reporting both nuclear and cytosolic localization. We detected PARN in cytoplasmic speckles and reconciled the diverging views in literature by identifying two PARN splice variants whose predicted localization is in agreement with those observations.

## 1. Introduction

Sulfur is one of six essential macronutrients plants absorb from the soil in large quantities to sustain growth and survival [1]. In the last decade, insufficient sulfate nutrition has been reported with increasing frequency in widely cultivated crops such as wheat, soybean and rapeseed [2,3,4]. Since prolonged sulfur depletion results in severe stunting and impaired resistance to biotic stress, this translates into significant losses in crop yield [5,6]. Understanding the mechanisms by which plants respond to and recover from sulfur deficiency is an essential step towards improving agricultural productivity.

Plants adapt to sulfur depletion by upregulating the expression of genes involved in sulfate uptake and reduction [6]. Additionally, the expression of negative regulators of glucosinolate biosynthesis is induced to prioritize sulfur usage for primary metabolism [7]. The reverse processes by which sulfur-deficient plants reshape their transcriptome upon sulfur resupply are, however, only poorly understood.

In resupply studies, Bielecka and coworkers identified so-called genuine sulfur-responsive transcripts that directly reflect the sulfur status of *Arabidopsis thaliana*. Most (30 out of 35) of those transcripts accumulate upon sulfur starvation and display rapid decay rates in the first hours after the resupply of the macronutrient [8]. Adopting an exponential decay model, the average half-life of those starvation-induced transcripts can be determined to amount to 2.3 hours during the recovery phase [8]. This is considerably shorter than the mean mRNA half-life of 5.9 hours measured in global studies of Arabidopsis mRNA stability under standard growth conditions [9,10]. Taken together, these findings suggest that during the recovery from sulfur limitation, the transcriptome is cleared of starvation-responsive mRNAs by active degradation rather than the regular turnover of transcripts. The clearance of stress-induced transcripts is also required for the recovery from high-light stress. In this context, the term “rapid recovery downregulation” has been coined. It describes the quick return of transcript abundance to pre-stress levels upon recovery [11]. Yet for both sulfur starvation and excess-light stress, it remains unclear which enzymes are mediating the targeted degradation of mRNAs upon recovery. 

In plants, a set of endonucleases, decapping enzymes and deadenylases govern the degradation of mRNA. While endonucleases cleave phosphodiester bonds within transcripts, decapping enzymes and deadenylases remove the methylguanine cap and the stabilizing poly(A) tail from the 5′ and 3′ ends of mRNAs [12,13,14,15]. The aforementioned enzymes are candidates to initiate the degradation of transcripts during the recovery from sulfur starvation. In this study, we focus on the role of the poly(A)-specific ribonuclease (*At*PARN) for the following reasons:

Unlike in *Schizosaccharomyces pombe* and *Caenorhabditis elegans*, PARN is essential for embryogenesis in *Arabidopsis thaliana*, indicating a unique role of PARN-mediated mRNA decay in higher plants [16]. *At*PARN hypomorphic mutants (*parn*) display diminished growth accompanied by an increased resistance to bacterial pathogens and a decreased tolerance towards osmotic stress [17,18,19,20]. These altered stress responses can be attributed to the accumulation of the phytohormone abscisic acid (ABA) in *parn* mutants, resulting from the disturbed equilibrium between polyadenylation and the deadenylation of specific stress-related transcripts [17,18,20,21]. Remarkably, sulfate and cysteine have recently been shown to trigger ABA biosynthesis in plants. Hence, the elevated ABA levels observed in *parn* mutants could be the result of increased sulfate assimilation [22,23]. 

Mammalian PARNs have been observed to preferentially degrade the poly(A) tails of transcripts harboring AU-rich signal elements (AREs) in their 3′ untranslated regions (UTRs). AREs range from 40 to 150 nucleotides in length and typically contain one or more AUUUA motifs within AU-rich sequence stretches [24,25,26]. The close evolutionary relationship between mammalian PARNs and *At*PARN suggests that *At*PARN might be recruited by AREs as well. In line with this assumption, *At*PARN was shown to target a specific subset of mRNAs rather than the entire mRNA population [16]. 

Here, we report that the AUUUA motif is present in the 3′UTRs of many transcripts induced by sulfur depletion, including the *O*-acetylserine cluster genes *SDI1*, *GGCT* and *SHM7* [27] as well as the high-affinity sulfur transporter genes *SULTR1;1* and *SULTR1;2*. This finding makes *At*PARN a potential candidate for the regulation of active mRNA degradation during the recovery from sulfur depletion. 

However, analysis of transcript stability by qRT-PCR in PARN hypomorphic mutants demonstrates that PARN is not required for the targeted degradation of sulfur deficiency-induced transcripts in Arabidopsis. To understand the biological role of PARN, we determine the subcellular localization of PARN and its antagonist, the poly(A) polymerase AGS1. To this end we image stable Arabidopsis lines expressing PARN-GFP or AGS1-GFP fusions under the control of the respective endogenous promoters. Unlike the predominantly nuclear localized AGS1, PARN accumulates in cytoplasmic speckles. So far, PARN has been observed to localize to processing bodies (p-bodies) when transiently expressed under the control of the 35S promoter in tobacco leaves [28]. This, however, contradicts earlier PARN localization studies in onion epidermal peels reporting a nuclear–cytoplasmic localization [16,29]. By detecting two PARN splice variants, which were bioinformatically predicted to localize to different cellular compartments, we offer an explanation for the diverging accounts of PARN localization in the literature. 

## 2. Results

Upon recovery from sulfur starvation, transcripts of many stress-induced genes are rapidly degraded. We reasoned that recognition signals embedded in those transcripts might provide specificity to the process and link them to the active degradation machinery of plants. AREs in the 3′UTRs of transcripts have been known to target mammalian mRNAs for rapid degradation by recruiting the deadenylase PARN [30]. This observation prompted us to search for AREs in the 3′UTRs of transcripts upregulated upon sulfur starvation.

### 2.1. The Sulfur-Responsive Transcripts SULTR1;1, SULTR1;2, SDI1, SHM7 and GGCT Contain ARE Sites 

**Figure 1:**
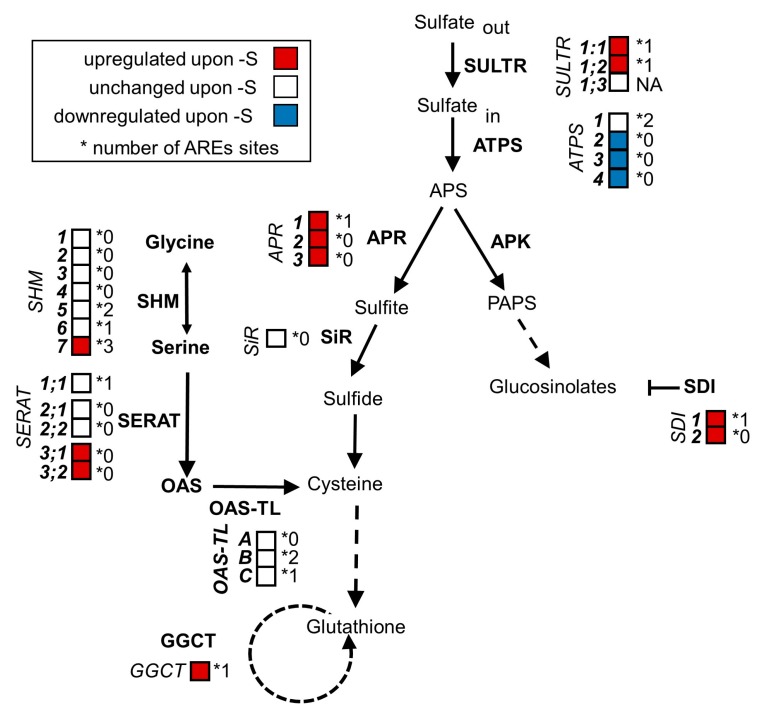
Plants regulate the transcription of genes involved in sulfate assimilation and glucosinolate biosynthesis in response to sulfur supply. When plants are exposed to sulfur limitation, they upregulate the expression of genes involved in sulfate uptake and assimilation. Simultaneously, the expression of genes implicated in the synthesis of sulfur-containing secondary metabolites is downregulated to prioritize sulfate usage for primary metabolites. In this scheme of plant sulfate metabolism, the transcript levels of genes (italics) encoding for enzymes mediating sulfate assimilation (bold) are indicated by a color code. Red and blue represent significant (p < 0.05; ≥2-fold) up- and downregulations under sulfur limitation. White represents no significant change in comparison to full nutrient supply. Many sulfur responsive transcripts harbor AU-rich signal elements (AREs) in their 3′ untranslated regions (UTRs). The number of AREs found in each 3′UTR is indicated next to the asterisk. (Transcript data from [31]).

We could identify AREs in many transcripts that are involved in sulfur metabolism and are upregulated upon sulfur starvation, including the *O*-acetylserine dependent cluster genes *SDI1*, *GGCT* and *SHM7* [27] as well as the high-affinity sulfate transporter genes *SULTR1;1* and *SULTR1;2* (Figure 1). In contrast, transcripts that were not induced upon sulfur starvation but are involved in sulfur metabolism did in many cases lack AREs (e.g., *SHM1-4*, *SERAT 2;1* and *SERAT2;2*, *OAS-TL A* and *SIR*). There was, however, no significant difference in ARE frequency between sulfur metabolism-related and general transcripts. Given the broad presence in mRNAs encoding for the sulfur metabolism pathway, we hypothesized that the degradation of sulfur-responsive transcripts upon the recovery from starvation might depend on PARN.

### 2.2. The Degradation of Sulfur Metabolism-Related Transcripts Is Independent of AtPARN

**Figure 2:**
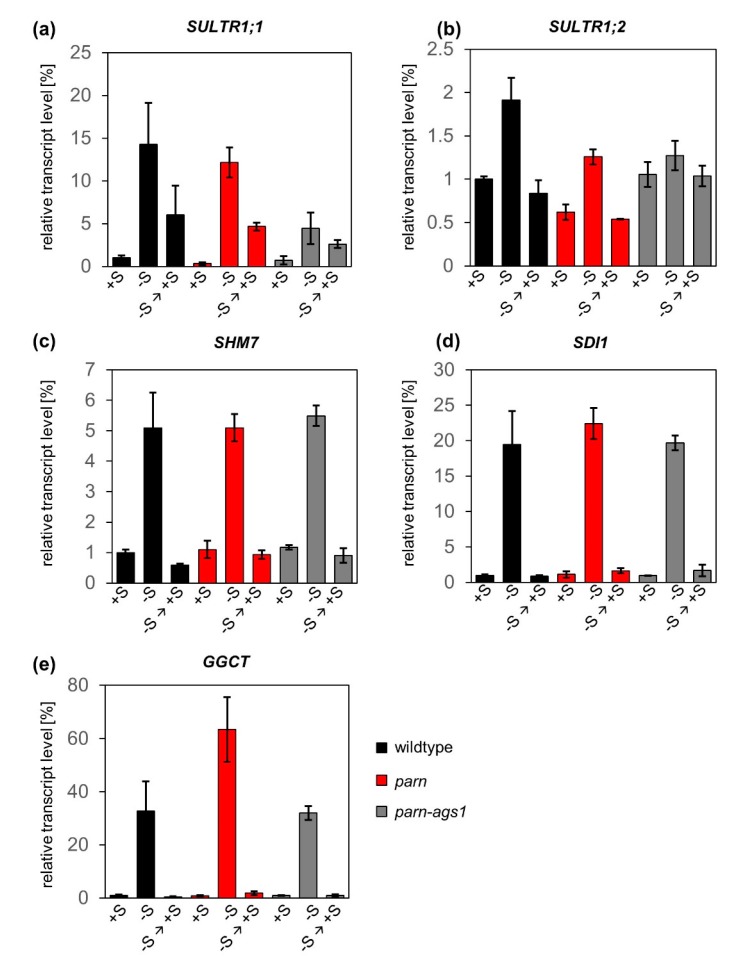
*At*PARN is not required for the degradation of sulfur starvation-induced transcripts upon the resupply of the macronutrient. Relative transcript levels of *SULTR1;1* (**a**), *SULTR1;2* (**b**), *SHM7* (**c**), *SDI1* (**d**) and *GGCT* (**e**) upon regular sulfur supply (+S, 500 μM), starvation (-S, two weeks at 0 μM) and recovery (-S → +S, 3 h at 500 μM) in roots of *parn* (red), *parn-ags1* (grey) and wildtype plants (black). Results were normalized to the expression values measured for wildtype plants under full nutrient supply. Bars represent standard errors (n = 3).

To determine whether PARN degrades starvation-induced transcripts after sulfur resupply in *Arabidopsis thaliana*, we depleted six-week-old hydroponically grown *parn, parn-ags1* and wildtype plants of sulfur. After two weeks of starvation (0 µM sulfur), the plants were transferred back to ½ Hoagland medium (500 µM sulfur) for three hours to allow recovery. Subsequently, the transcript levels of the sulfur-starvation marker genes *SULTR1;1*, *SULTR1;2*, *SDI1*, *SHM7* and *GGCT* were assessed via qRT-PCR in roots (Figure 2).

The *AGS1* gene encodes for a poly(A) polymerase, which acts as an antagonist of PARN. Since all of the known *parn* phenotypes are suppressed by loss-of-function mutations in *AGS1* [20,21], we expected the *parn-ags1* double mutants to reset their transcriptome to pre-starvation conditions just as readily as wildtype plants.

As suggested by publicly available microarray data [17,18], the transcript levels of *SULTR1;1, SULTR1;2, SHM7, SDI1* and *GGCT* did not differ considerably between wildtype plants and *parn* hypomorphic or *parn-ags1* double mutants under full nutrient supply. Upon starvation, a clear upregulation of the previously mentioned transcripts was observed in all genotypes. The strongest induction was measured for *GGCT* (33-fold for wildtype, 63-fold for *parn* and 32-fold for *parn-ags1*), whereas the transcript levels of *SULTR1;2* increased to a lesser extent (2-fold for wildtype, 2-fold for *parn* and 1.3-fold for *parn-ags1*). This is well in line with published transcript data from sulfur-starved wildtype plants [31,32] and supports the validity of the nutrient starvation conditions used. After sulfate resupply, the abundance of the five sulfur starvation marker transcripts decreased rapidly, not only in wildtype plants, but also in *parn* and *parn-ags1* mutants. With the exception of *SULTR1;1*, three hours of recovery were sufficient for the transcript levels to return to pre-starvation conditions. When this single time point was used as a basis for a rough estimation of transcript half-life, the measurements taken for wildtype plants indicated transcript half-lives of 151 minutes for *SULTR1;2*, 144 min for *SULTR1;1*, 58 min for *SHM7*, 40 min for *SDI1* and 29 min for *GGCT*. 

Taken together, these results indicate that *parn* and *parn-ags1* mutants clear their transcriptomes of surplus sulfur-responsive transcripts just as readily as wildtype plants. This finding excludes a significant function of PARN and its antagonist AGS1 in the clearance of sulfur starvation-induced transcripts. Furthermore, it puts a note of caution on the identification of functional ARE sites in plants based on the currently available prediction tools (or data on mammalian ARE sites). 

### 2.3. PARN Accumulates in Cytoplasmic Speckles

The subcellular localization of enzymes provides the physiological context for their activity and determines their access to substrates and interaction partners [33]. Therefore, the identification of the subcellular localization of PARN and its antagonist—the poly(A) polymerase AGS1—is critical to understanding the biological role of the PARN-AGS1 mRNA degradation system.

**Figure 3:**
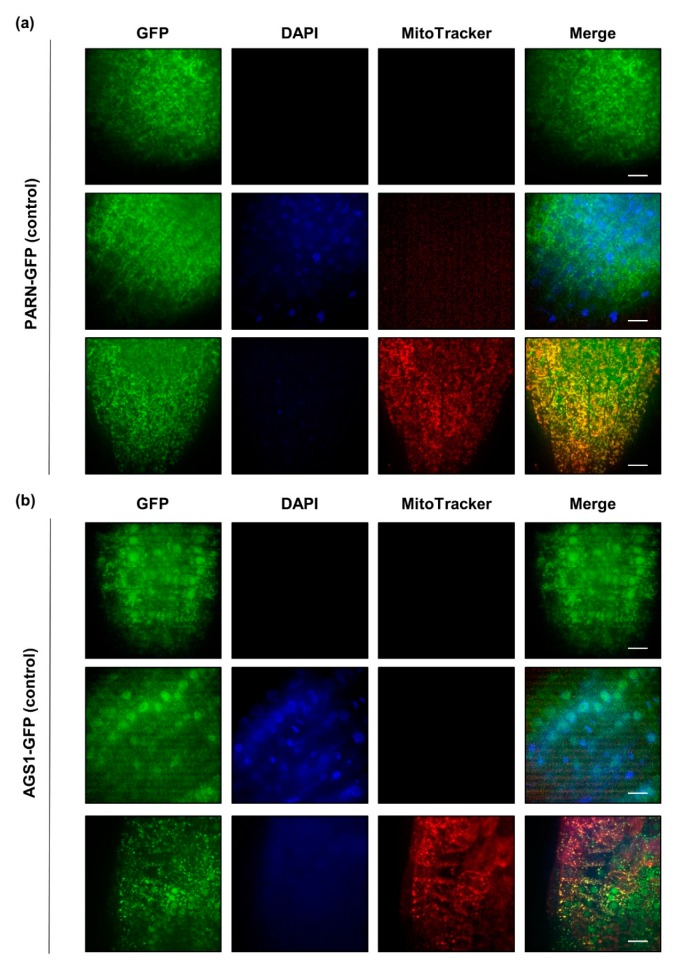
Under full nutrient supply, PARN-GFP (**a**) localizes to cytoplasmic speckles, whereas AGS1-GFP (**b**) is confined to the nucleus. Roots (tip and elongation zones) of ten-day-old PARN-GFP and AGS1-GFP seedlings grown under control conditions were left untreated (first row) or incubated with DAPI (second row) and MitoTracker Orange (third row). Each column represents a different channel (GFP, DAPI, MitoTracker). The last column shows a merge of all channels. Pictures are the result of maximum intensity z-projections of slices in a z-stack. Scale bar 10 µm.

In order to elucidate the subcellular localization of PARN and AGS1, we used stable transgenic lines expressing either PARN-GFP or AGS1-GFP under the control of their endogenous promoters [21]. These lines were germinated on agar-medium in the presence and absence of sulfur. Root sections from the tip to the elongation zone of ten-day-old seedlings were imaged for GFP signals after incubation, with dyes staining the mitochondria (MitoTracker, 100 nM for 15 min) and the nucleus (DAPI, 2 µg mL^−1^ for 15 min). Under full nutrient supply, PARN-GFP localized exclusively to cytoplasmic speckles that were partly, but not entirely, overlapping with the mitochondrial signal. AGS1-GFP was found in cytosolic speckles, as well as in the nucleus, where it was evenly distributed (Figure 3). In order to demonstrate that the observed GFP signals were not bleed-through signals from the DAPI or the MitoTracker channel, wildtype plants were stained with both dyes (Figure S1). Since AGS1 is an antagonist of PARN, we hypothesized that PARN might also localize to the nucleus under certain conditions.

Indeed, when the growth media were prepared without sulfur, in rare cases the subcellular localization of PARN-GFP shifted from cytoplasmic speckles to the nucleus (Figure S2a). AGS1-GFP, on the other hand, did not display any changes in localization upon nutrient starvation. To determine whether the starvation-induced relocalization of PARN to the nucleus was sulfur-specific or a general adaptation to nutrient starvation, the experiment was repeated with seedlings depleted of nitrogen (Figure S2b). Both nitrogen and sulfur are important macronutrients required for the synthesis of essential amino acids. As observed for sulfur depletion, nitrogen starvation induced a relocalization of PARN to the nucleus at a comparably low frequency. Similarly, reductive stress induced by 30 min of 10 mM dithiothreitol (DTT) caused PARN to shift its subcellular localization from cytosolic speckles to the nucleus (Figure S2c). Similar to nutrient starvation, the reductive stress treatment did not induce a comprehensive relocalization. However, the occasional relocalization of PARN-GFP under the applied stress conditions was never observed for the AGS1-GFP fusion under identical conditions (Figure S3).

### 2.4. PARN is Encoded for by Two Alternative Splice Variants Predicted to Localize to Different Cellular Compartments

**Figure 4:**
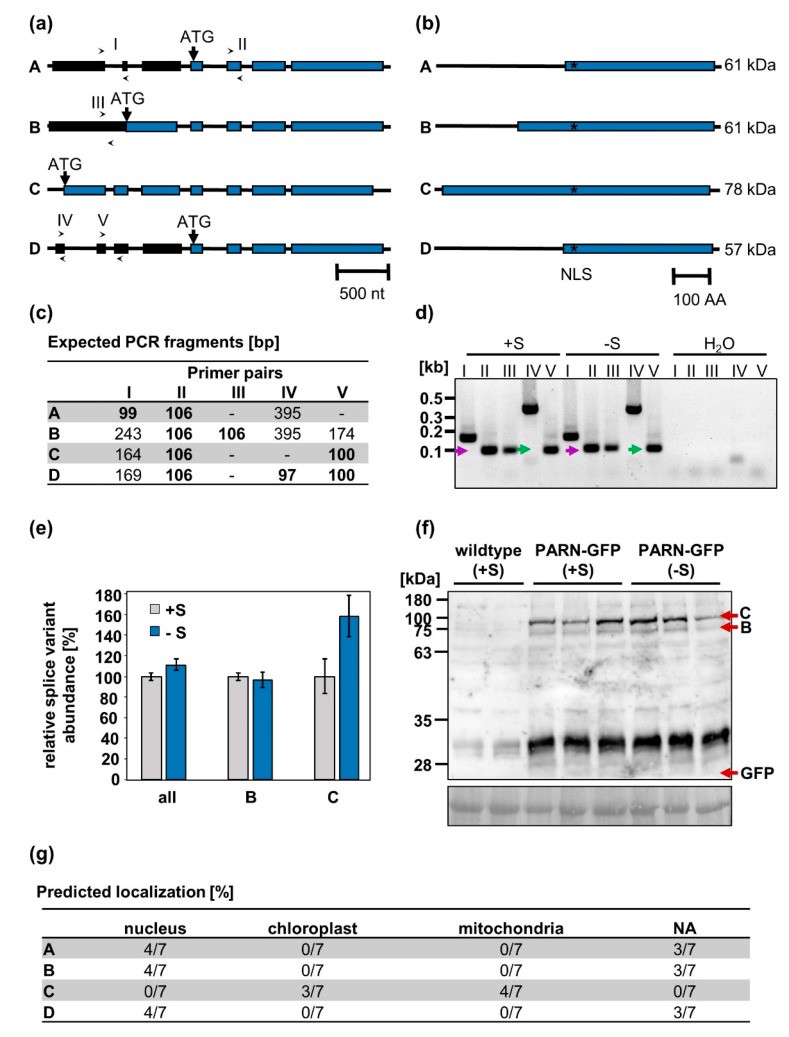
*At*PARN is encoded for by four splice variants. (**a**) Schematic structure of splice variants A–D. Their presence was verified with primer pairs I–V (indicated by arrows). While black boxes represent untranslated exons, blue boxes indicate translated exons. Introns are represented by black lines. (**b**) Proteins encoded for by transcripts A–D. The predicted nuclear localization signal (NLS) is marked with an asterisk. (**c**) Expected PCR products for primer pairs I–V in the presence of splice variants A–D. Most primer pairs may generate several amplicons of differing lengths, depending on which isoforms are actually present. Due to the extremely short annealing/extension step however, only the shortest amplicons (bold) are expected to be produced. (**d**) qRT-PCR products generated using primers I–V on cDNA from roots of starved (-S, 0 µM sulfur) and non-starved (+S, 500 µM sulfur) wildtype plants. As a negative control, the cDNA was substituted with H_2_O. (**e**) Relative abundance of splice variants B and C as well as total PARN transcripts under full nutrient supply and sulfur starvation in wildtype roots. (**f**) Immunodetection of the PARN-GFP isoforms B and C in protein extracts from sulfur-starved and non-starved seedlings with a polyclonal rabbit α-GFP antibody (# A-6455, Thermo Fisher Scientific). Amido black-stained protein served as the loading control. (**g**) Localization of isoforms A–D as predicted by seven independent algorithms (see 4.7). Since the cytosol is the default localization of a protein, cytosolic proteins will not yield any prediction by the aforementioned algorithms (denoted by “N.A.”).

One possible explanation for the dual localization of PARN observed in our experiments is the existence of alternative PARN splice isoforms encoding for different protein variants confined to distinct cellular compartments. The Arabidopsis Information Resource (TAIR) provides sequences of four *At*PARN splice variants, A–D (Figure 4a). Since the splice variants A and D give rise to the same protein, the four splice variants encode for three distinct protein species. These species differ only in the length of their N-terminus (Figure 4b). Since the N-terminus is an important determinant of subcellular protein localization, the localization of the isoforms A–D was predicted by seven algorithms. While the splice variants A and B were predicted to localize to the nucleus or the cytoplasm (denoted as “N.A.”), splice variant C was predicted to be confined to the mitochondria or the chloroplasts (Figure 4g). 

To determine which of the four *PARN* transcripts were present in planta under full nutrient supply and sulfur starvation, the primers I–V detecting the splice variants A–D (Figure 4a) were used for a qRT-PCR analysis of cDNA extracted from starved and non-starved wildtype plants. By comparing the expected and observed fragments produced by the isoform-specific primers, the presence of the splice isoforms B and C could be verified in vivo. Isoforms A and D, however, were not detected. These results were further corroborated by the immunodetection of the splice variants B and C, but not A and D, in protein extracts from 10-day-old seedlings (Figure 4f). Remarkably, no difference in patterns between the seedlings grown under sulfur deficiency and full nutrient supply could be observed. Under both conditions, isoform C was expressed at an approximately 4-fold higher level than isoform B. No free GFP was detected, indicating that the nuclear GFP signal observed in PARN-GFP roots cannot be attributed to cleaved fluorophores, but is indeed an authentic GFP-PARN signal. Furthermore, the qRT-PCR analysis revealed that neither the total PARN transcript levels nor the ratio of isoform B to isoform C changed significantly when comparing full and limited nutrient supply (Figure 4e). The relative abundance of isoform B was calculated using the primer pair IV, whereas the primer pair V was used to determine the relative abundance of isoform C. Primer pair II was used to quantify the total PARN transcript level.

## 3. Discussion

Plants as sessile organisms rely on transcriptional reprogramming to adapt to a constantly changing macronutrient supply. When exposed to sulfur limitation, they upregulate sulfate assimilation pathways, resulting in an accumulation of transcripts encoding for sulfur deficiency marker genes [1]. Upon cessation of the stress, however, those transcripts are subjected to rapid recovery downregulation [8,11]. In eukaryotes, mRNA decay is generally initiated by the deadenylation of transcripts [25]. As mutations in the poly(A) ribonuclease *At*PARN have been linked to altered abiotic stress responses in *Arabidopsis thaliana* [17,18,19], this manuscript investigated a few aspects of the role of PARN in the recovery from sulfur starvation.

### 3.1. The Degradation of Sulfur Starvation-Induced Genes during the Recovery from Starvation is Independent of AtPARN 

Although we identified putative PARN-recruiting AREs [25] in several sulfur starvation-induced transcripts, PARN is not required for their degradation upon recovery from starvation. When mutants of *parn* and its antagonist *ags1* were subjected to sulfur depletion followed by resupply, they degraded surplus sulfur-induced transcripts just as effectively as wildtype plants (Figure 2). The mRNA half-life estimations inferred from the observed transcript degradation rates of *GGCT*, *SDI1* and *SHM7* amounted to less than an hour. Since those calculations were based on only one time point, they can only provide a rough estimate of the upper limit of the mRNA half-life. In Arabidopsis, the average mRNA half-life is estimated to be in the order of several hours [10]. This indicates that even though PARN is not mediating the degradation of *GGCT*, *SDI1* and *SHM7*, other active mRNA degradation enzymes might act on those transcripts upon sulfur resupply. The sulfur-responsive transcripts might, for instance, be degraded by the cytoplasmic exoribonuclease XRN4. XRN4 has been shown to degrade the mRNA of heat shock factor HSFA2 and thereby represses heat stress responses after the return to normal temperature [34]. The fact that XRN4 targets specific transcripts involved in the response to abiotic and biotic stimuli [35] supports the notion that XRN4, rather than PARN, might be involved in the recovery from sulfur starvation in *Arabidopsis thaliana.*

### 3.2. The Presence of Two Alternative PARN Splice Variants Reconciles the Diverging Views on PARN Localization in the Literature

We selected PARN as a candidate for rapid transcript degradation upon sulfur resupply because we identified putative PARN-recruiting AREs in mRNAs upregulated upon sulfur starvation. Transcripts harboring AREs are known to localize to the cytosolic sites of mRNA degradation, termed processing-bodies (p-bodies), where they are rapidly degraded [36]. 

According to translational fusions with GFP, PARN localizes to p-bodies when it is transiently expressed under the control of the 35S promoter in tobacco leaves [28]. This finding is discussed controversially in the literature, since it contradicts earlier PARN localization studies in onion epidermal peels, reporting a nuclear–cytoplasmic localization [16,29]. Both observations do however agree with the predominantly nuclear–cytosolic localization of PARN reported for metazoans. When *At*PARN-GFP expression is driven by the native PARN promoter, the fusion protein localizes to the mitochondria, indicating a unique function of PARN in higher plants [20,21]. 

Here we made use of stable transgenic PARN-GFP lines to show that under optimal nutrient supply, PARN localizes to cytoplasmic speckles. Unlike previously reported by Hirayama and co-workers [21], we did not observe full colocalization of those speckles with the mitochondria. Our findings suggest that PARN might localize to the mitochondria, but a considerable portion of the observed PARN-GFP signal is localized to p-bodies [28]. Since p-bodies are involved in the degradation and translational arrest of transcripts during development and the adaptation to stress, this subcellular localization agrees with the biological function of PARN observed in mammals. 

Furthermore, we found that mineral nutrient deficiency and reductive stress in rare cases induces a dynamic delocalization of PARN from cytoplasmic speckles to the nucleus. The fact that this delocalization was observed under several stress conditions points to a general cellular mechanism. Although the function of PARN in the nucleus remains unknown, the stress-induced delocalization system we describe here opens new avenues to study the function of nuclear-localized PARN.

A potential mechanism for the stress-induced delocalization of the PARN protein is the existence of different PARN splice variants as evidenced by The Arabidopsis Information Resource (TAIR). These variants encode for proteins that differ only in the length of their N-terminus. Whereas the full-length *At*PARN splice variant carries an N-terminal extension that distinguishes *At*PARN from putative animal homologs [20], the shorter *At*PARN variants lack this non-conserved N-terminus. When subjected to intracellular targeting algorithms, the different *At*PARN splice variants are bioinformatically predicted to localize to distinct subcellular compartments. It is worth noting that three of the seven applied prediction algorithms (iPSORT, TargetP and Predotar) focus on N-terminal sorting signals to determine the localization of proteins [37,38,39]. Thus, their predictions are biased towards mitochondrial and chloroplastic localizations. While the full-length protein is predicted to localize to the mitochondria, the shorter variants are thought to localize to the nucleus and the cytoplasm. We detected two out of the four *At*PARN splice variants in roots of wildtype Arabidopsis plants. These splice variants are predicted to localize to the nucleus or the cytoplasm and the mitochondria. 

Profiling of *PARN* transcripts from roots revealed that both splice forms were present at similar levels under optimal or sulfur-depleted conditions. Hence, changes in the transcription or processing of both variants cannot directly explain the sulfur limitation-induced delocalization of PARN in roots. However, both splice variants were present in roots under full nutrient supply as well as starvation conditions, which potentially enables stress-induced differential loading of the transcripts to ribosomes. Indeed, in yeast and mammalian somatic cells, thousands of untranslated mRNAs were shown to be targeted to p-bodies, where they are translationally repressed, suggesting that p-bodies provide a reservoir for quick adaptation of gene expression [40]. Similarly, plant p-bodies might act as reservoirs for the two *PARN* transcript variants, enabling plants to react to stresses without the delay caused by a transcriptional regulation of *PARN*. 

Immunodetection of *At*PARN under standard growth conditions and sulfur depletion, however, revealed no shift in the ratio between isoform B and C on the protein level. Most likely, the expression of isoform B is not sufficient to induce nuclear localization but requires stress-induced post-translational modifications of PARN or the binding of PARN to interaction partners. Both mechanisms have previously been described for other proteins. The Arabidopsis leucine zipper transcription factor VIP1, for instance, is dephosphorylated upon mechanical and hypo-osmotic stress and subsequently changes its localization from the cytosol to the nucleus [41]. In mammalian cells, RNA-binding proteins move from the cytoplasm to the nucleus in response to accelerated mRNA decay associated with cellular stress. In some cases, this delocalization is mediated via direct interaction of those proteins with the nuclear transport machinery. In other cases, interactions with proteins containing NLS are responsible for the import into the nucleus. On the other hand, PARN might be sequestered in the cytosol via interaction with cytosolic proteins. In accordance with this hypothesis, the BioAnalyticResource Tool Arabidopsis Interaction Viewer predicts PARN to interact with two cytosolic proteins (AT5G47010 and AT2G39260) required for nonsense-mediated mRNA decay. The mechanism of PARN delocalization will be the subject of further studies. 

## 4. Materials and Methods 

### 4.1. Plant Material and Growth Conditions

All work was performed with *Arabidopsis thaliana* ecotype Columbia-0 (Col-0). The transgenic *parn* knockdown, *parn-ags1* double mutant, PARN-GFP and AGS1-GFP lines are characterized in [19,21]. All experiments except the subcellular localization study and the sulfur resupply assay (described below) were conducted with plants grown under short-day conditions (8.5 h light, 100 μE light photon flux density, 24 °C by day, 18 °C by night and 50% humidity) on a medium containing one half soil and one half substrate 2 (Klasmann-Deilmann, Germany).

### 4.2. Sulfur Resupply Assay

In order to determine the role of *At*PARN in the recovery from sulfur starvation, *parn*, *parn-ags1* and wildtype seeds were surface-sterilized with 70% (v/v) ethanol for 5 min followed by 6% sodium hypochlorite for 3 min and a second wash with 70% (v/v) ethanol for 5 min. Afterwards, the seeds were washed trice with ddH_2_O. Individual seeds were placed in microcentrifuge tubes containing ½ Hoagland medium (0.5 mM KH_2_PO_4_, 0.05 µM (NH_4_)_6_Mo_7_O_24_ *·* 4 H_2_O, 0.5 mM MgSO_4_/MgCl_2_, 0.15 µM CuSO_4_ *·* 5 H_2_O, 2.5 mM Ca(NO_3_)_2_ *·* 4 H_2_O, 1.9 µM ZnSO_4_ *·* 7 H_2_O, 2.5 mM KNO_3_, 10 µM NaCl, 2.25 µM MnCl_2_ *·* 4 H_2_O, 25 µM H_3_BO_3_, 40 µM Fe-EDTA; pH 5.8) supplemented with 0.6% (w/v) agar. Subsequently, the tubes were inserted in standard 1 ml pipette tip racks. Plants were stratified at 4 °C for three days before being germinated in a short-day growth cabinet. After two weeks, individual plants were transferred to 6 liter boxes containing ½ Hoagland medium. After an additional two weeks of growth on full medium, a subset of the plants was starved for sulfur by replacing MgSO_4_ in the Hoagland medium with MgCl_2_. The control group continued to receive full medium. Starvation lasted for two weeks, with media being exchanged on a weekly basis. Subsequently, root and shoot material were collected and snap-frozen in liquid nitrogen. 

### 4.3. Genotyping by PCR

In order to identify the transgenic plants, gDNA was extracted from 50–100 mg Arabidopsis leaf material. The fresh tissue of four-week-old plants was ground with a plastic pestle for 10–15 sec. Subsequently, 400 µL Edwards buffer (200 mM Tris/HCl, 25 mM EDTA, 250 mM NaCl, 0.5% SDS) were added and the mixture was vortexed for 5 sec. After centrifugation at 13.000 rpm for 5 min at room temperature, 300 µL of supernatant were transferred to a fresh microfuge tube. 300 µL 100% isopropanol were added and the mixture was left to incubate for two minutes at room temperature. After centrifugation at 13,000 rpm for 10 min at room temperature, the supernatant was discarded and the pellet was washed with 700 µL 70% ethanol. After a final centrifugation step at 13,000 rpm for 10 min, the ethanol was discarded and the DNA pellet was resuspended in 40 µL ddH_2_O. Subsequently, 20 ng of the harvested gDNA were used for PCR reactions performed with the GoTaq^®^ Green Master Mix (Promega) and specific primers (see Appendix A Table A1). 

### 4.4. Quantifying Gene Expression by qRT-PCR

To analyze the transcript levels of sulfur starvation-induced genes, total RNA was extracted from frozen leaf and root material using the peqGOLD total RNA kit (PeqLab). Subsequently, total RNA was transcribed into complementary DNA (cDNA) with the RevertAid H Minus First Strand cDNA Synthesis Kit using oligo(dT) primers (Thermo Fisher Scientific). All reactions were conducted according to the supplier’s protocol. The cDNA was analyzed by qRT-PCR with the SqPCRBIO SyGreen Mix Lo-ROX (Nippon Genetics Europe GmbH) and *TIP41* (AT4G34270, [42]) and *PP2A* (AT1G69960, [43]) as reference genes. The corresponding primer sequences are listed in Table A3 of the Appendix A. Data was analyzed via the Rotor-Gene Q Series Software. 

In order to quantify the amount of transcripts that encode for alternatively spliced forms of *At*PARN, primers that discriminated between the mRNA models A–D were designed and used for qRT-PCR (see Table A2 of the Appendix A for sequences). The four models encode for the cDNA clones AK227465 and AB223028 (A), AB223029 (B), AB19466 (C) and AB223027 (D). To ensure that each primer pair produced only the shortest possible fragment, the annealing time was reduced to 20 sec. As a quality control measure, a melting curve (ramp from 63 °C–95 °C rising by 1 °C per step) was recorded. Additionally, the qRT-PCR products were visualized on agarose gels (0.8% agarose in 40 mM Tris, 20 mM acetic acid, 1 mM EDTA, pH 8.0).

The shares of the individual isoforms A–D of the total transcript amount of *At*PARN were calculated based on the ∆∆CT method [44].

### 4.5. Immunodetection of PARN-GFP

Total proteins were extracted from 10-day-old PARN-GFP and wildtype seedlings sown on ½ Hoagland medium supplemented with 0.8% agarose and either 0 µM (-S) or 500 µM (+S) sulfate. After extraction in 80 mM Tris-HCl (pH 7.5), 300 mM NaCl, 1 mM EDTA, 1 mM PMSF, 10 mM DTT, 1% TritonX and 1 tablet EDTA-free protease Inhibitor cocktail per 50 ml (Roche), the samples were subjected to discontinuous SDS–PAGE in Mini-ProteanTM II cells (BioRad), followed by immunoblotting with a polyclonal rabbit α-GFP antibody (# A-6455, Thermo Fisher Scientific) diluted 1:5000 in TBS-T (50 mM Tris pH 7.6, 150 mM NaCl, 0.05% Tween-20). After blocking for 1 h with 5% BSA in TBS-T, the blot was washed trice with TBS-T before the addition of the primary antibody, which was left to incubate overnight at 4 °C. Subsequently, the blot was washed trice with TBS-T and the secondary horseradish peroxidase-linked anti-rabbit antibody (#AS10 852, Agrisera) diluted 25,000-fold in TBS-T was left to incubate for 1 h. Membranes were developed using SuperSignal West Dura Extended Duration Substrate (Thermo Fisher Scientific) according to the manufacturer’s protocol. The resulting signals were recorded using the ImageQuant LAS 4000 (GE Healthcare).

### 4.6. Subcellular Localization 

To assess the subcellular localization of *At*PARN and its antagonist AGS1, *At*PARN-GFP and AGS1-GFP seeds were sterilized as described previously and sown on ½ Hoagland medium supplemented with 0.8% agarose. The plants were stratified at 4 °C in the dark for three days before they were transferred to long-day conditions (16 h light, 100 μE light photon flux density, 24 °C by day, 18 °C by night and 50% humidity). In order to visualize the mitochondria, 10-day-old seedlings were incubated with 100 nM MitoTracker™ Orange CMTMRos (Thermo Fisher Scientific) in ½ Hoagland medium for 15 min as described in [21]. For nuclear staining, samples were incubated for 15 min with 2 µg ml^−1^ DAPI (Sigma-Aldrich) in ½ Hoagland medium supplemented with 1:20,000 Triton-X. Samples grown on -S plates were incubated in staining solutions prepared with -S ½ Hoagland medium. For the DTT treatment, seedlings were floated for 30 min in ½ Hoagland medium supplemented with 10 mM DTT before staining with MitoTracker or DAPI. Subsequently, the roots were separated from the seedlings and imaged with a Nikon automated Ti inverted microscope equipped with a Yokagawa CSU-X1 confocal scanning unit, a Hamamatsu C9100-02 EMCCD camera and a Nikon Plan Apo VC 100x NA 1.4 oil immersion objective (Nikon). Images were taken as z-stacks with an approximate thickness of 1 µm. Images were taken in three different channels (DAPI: 405 nm/445 nm; GFP 488 nm/527 nm; MitoTracker 561 nm/615 nm). Additionally, a brightfield image was recorded. The resulting z-stacks were processed with the open-source image analysis software Fiji [45]. For each channel, a maximum intensity z-projection image was calculated. Subsequently, the background fluorescence intensity was measured and subtracted for each channel. 

### 4.7. Identification and Functional Annotation of Genes with mRNA Destabilizing Motifs

Sequence data for all known 3′UTRs of cytosolic mRNAs was downloaded from The Arabidopsis Information Resource (TAIR) (TAIR10 blastsets, TAIR10_3_utr_20101028, as of 10.11.2010). Subsequently, pattern match algorithms were devised to search the sequence strings for the occurrence of the core sequence “AUUUA”, characteristic for AU-rich elements. 

### 4.8. Prediction of Subcellular Protein Localization

The subcellular localization of *At*PARN was predicted based on its amino acid sequence. For that purpose, several bioinformatical tools, including Predotar [37], iPSORT [38], Target [39], SherLock2 [46], BaCelLo [47], WoLF PSORT [48] and YLoc [49] were applied.

The presence of nuclear localization signals was predicted with the public NLS db server (to be found at www.rostlab.org/services/nlsdb/, as of 13.04.2018).

## Supplementary Materials

The following are available online at https://www.mdpi.com/2223-7747/8/10/380/s1, Figure S1: Wildtype seedlings stained with DAPI and MitoTracker display no signal in the GFP channel, Figure S2: AGS1-GFP does not change its subcellular localization upon nutrient starvation, Figure S3: AGS1-GFP does not change its subcellular localization upon nutrient starvation.

## Appendix A

**Table A1:** Primers used to genotype transgenic plant lines.

Allele	TAIR Identifier	Sequence
*ACTIN7*	AT5G09810	CAACCGGTATTGTGCTCGATTC
		GAGTGAGTCTGTGAGATCCCG
*AGS1*	AT2G17580	CTAGCAAATTCGACAGCTTTGC
		ATTTTAGAGGTTATTCTCCAATATGG
*AGS1* mutated	AT2G17580	ATTTTAGAGGTTATTCTCCAATATGA
*AtPARN*	AT1G55870	CTGATTCAGATTCCGACAAGGA
		CTTTGCCTCCTTCTGTGAAAAG
*AtPARN* mutated	AT1G55870	GTATACTGATTCAGATTCCGACAAA
*GFP*	N.A.	GCGGATCTTGAAGTTGGCC
*GFP*	N.A.	CGACGGCAACTACAAGACC

**Table A2:** Primers used to identify *At*PARN splice variants.

Primer	Sequence
I	CCCTTTCGTTGGGATTCTCG
	GTATGCAGGTGTTGAAATCAAAC
II	CACGAATTTCTCAGCTGTTGAAG
	TGCTTCAGAGAAAAAGCTGATCG
III	CTACCCGTTAGTCTCTCTTTC
	GACGAGGAAATACAAAGAAATTGTG
IV	GCAAAACCTAAAAATGGTCGTTTG
	CGAGAATCCCAACGAAAGGG
V	CCCTTTCGTTGGGATTCTCG
	CATGAGCTGGTGGATCAAATG

**Table A3:** Primers used to assess abundance of genuine sulfur-responsive transcripts.

Allele	TAIR Identifier	Sequence
*GGCT*	AT1G44790	CCGGAGCTATTTGCTGGGGTG
		GTCGTATTCACACTCTCTTCGTTCC
*PP2A*	AT1G69960	CTTCTCGCTCCAGTAATGGGATCC
		GCTTGGTCGACTATCGGAATGAGAG
*SDI1*	AT5G48850	CCCTTGACAATGTCCTCATCG
		GCTTCTCCTTGATAGATCTGCC
*SHM7*	AT1G36370	CTATACAGCCTCGGGTTGTCATTG
		AACTAACGTCATTACATACACATCTTG
*SULTR1;1*	AT5G04590	GTCCGGGACTATTAATCCC
		CGTACCCCATGCTCAGCG
*SULTR1;2*	AT1G78000	GGATCCAGAGATGGCTACATGA
		TCGATGTCCGTAACAGGTGAC
*TIP41*	AT3G54000	GATGAGGCACCAACTGTTCTTCGTG
		CTGACTGATGGAGCTCGGGTCG

## References

[1] Kopriva S., Mugford S.G., Baraniecka P., Lee B.R., Matthewman C.A., Koprivova A. (2012). Control of sulfur partitioning between primary and secondary metabolism in Arabidopsis. Front. Plant Sci..

[2] Jackson T.L., Baker G.W., Wilks F.R., Popov V.A., Mathur J., Benfey P.N. (2015). Large cellular inclusions accumulate in Arabidopsis roots exposed to low-sulfur conditions. Plant Physiol..

[3] Zhao F.J., Hawkesford M.J., McGrath S.P. (1999). Sulphur assimilation and effects on yield and quality of wheat. J. Cereal. Sci.

[4] Ding Y., Zhou X., Zuo L., Wang H., Yu D. (2016). Identification and functional characterization of the sulfate transporter gene *GmSULTR1;2b* in soybean. BMC Genom..

[5] Lewandowska M., Sirko A. (2008). Recent advances in understanding plant response to sulfur-deficiency stress. Acta Biochim. Pol..

[6] Nikiforova V., Freitag J., Kempa S., Adamik M., Hesse H., Hoefgen R. (2003). Transcriptome analysis of sulfur depletion in *Arabidopsis thaliana*: Interlacing of biosynthetic pathways provides response specificity. Plant J..

[7] Maruyama-Nakashita A. (2017). Metabolic changes sustain the plant life in low-sulfur environments. Curr. Opin. Plant Biol..

[8] Bielecka M., Watanabe M., Morcuende R., Scheible W.-R., Hawkesford M.J., Hesse H., Hoefgen R. (2015). Transcriptome and metabolome analysis of plant sulfate starvation and resupply provides novel information on transcriptional regulation of metabolism associated with sulfur, nitrogen and phosphorus nutritional responses in Arabidopsis. Front. Plant Sci..

[9] Crisp P.A., Ganguly D., Eichten S.R., Borevitz J.O., Pogson B.J. (2016). Reconsidering plant memory: Intersections between stress recovery, RNA turnover, and epigenetics. Sci. Adv..

[10] Narsai R., Howell K.A., Millar A.H., O’Toole N., Small I., Whelan J. (2007). Genome-wide analysis of mRNA decay rates and their determinants in *Arabidopsis thaliana*. Plant Cell.

[11] Crisp P.A., Ganguly D., Smith A.B., Murray K.D., Estavillo G.M., Searle I.R., Ford E., Bogdanović O., Lister R., Borevitz J.O. (2017). Rapid recovery gene downregulation during excess-light stress and recovery in Arabidopsis. Plant Cell.

[12] Nakaminami K., Matsui A., Shinozaki K., Seki M. (2012). RNA regulation in plant abiotic stress responses. Biochim. Biophys. Acta.

[13] Christie M., Brosnan C.A., Rothnagel J.A., Carroll B.J. (2011). RNA decay and RNA silencing in plants: Competition or collaboration?. Front. Plant Sci..

[14] Belostotsky D.A., Sieburth L.E. (2009). Kill the messenger: mRNA decay and plant development. Curr. Opin. Plant Biol..

[15] Chiba Y., Green P.J. (2009). mRNA degradation machinery in plants. J. Plant Biol..

[16] Reverdatto S.V., Dutko J.A., Chekanova J.A., Hamilton D.A., Belostotsky D.A. (2004). mRNA deadenylation by PARN is essential for embryogenesis in higher plants. RNA.

[17] Nishimura N., Kitahata N., Seki M., Narusaka Y., Narusaka M., Kuromori T., Asami T., Shinozaki K., Hirayama T. (2005). Analysis of *ABA hypersensitive germination 2* revealed the pivotal functions of PARN in stress response in Arabidopsis. Plant J..

[18] Nishimura N., Okamoto M., Narusaka M., Yasuda M., Nakashita H., Shinozaki K., Narusaka Y., Hirayama T. (2009). *ABA hypersensitive germination 2-1* causes the activation of both abscisic acid and salicylic acid responses in Arabidopsis. Plant Cell Physiol..

[19] Nishimura N., Yoshida T., Murayama M., Asami T., Shinozaki K., Hirayama T. (2004). Isolation and characterization of novel mutants affecting the abscisic acid sensitivity of Arabidopsis germination and seedling growth. Plant Cell Physiol..

[20] Hirayama T. (2014). A unique system for regulating mitochondrial mRNA poly(A) status and stability in plants. Plant Singal. Behav..

[21] Hirayama T., Matsuura T., Ushiyama S., Narusaka M., Kurihara Y., Yasuda M., Ohtani M., Seki M., Demura T., Nakashita H. (2013). A poly(A)-specific ribonuclease directly regulates the poly(A) status of mitochondrial mRNA in Arabidopsis. Nat. Commun..

[22] Malcheska F., Ahmad A., Batool S., Muller H.M., Ludwig-Muller J., Kreuzwieser J., Randewig D., Hansch R., Mendel R.R., Hell R. (2017). Drought-enhanced xylem sap sulfate closes stomata by affecting ALMT12 and guard cell ABA synthesis. Plant Physiol..

[23] Batool S., Uslu V.V., Rajab H., Ahmad N., Waadt R., Geiger D., Malagoli M., Hedrich R., Rennenberg H., Herschbach C. (2018). Sulfate is incorporated into cysteine to trigger ABA production and stomata closure. Plant Cell.

[24] Lai W.S., Kennington E.A., Blackshear P.J. (2003). Tristetraprolin and its family members can promote the cell-free deadenylation of AU-rich element-containing mRNAs by poly(A) ribonuclease. Mol. Cell. Biol..

[25] Chen C.Y., Shyu A.B. (2011). Mechanisms of deadenylation-dependent decay. Wiley Interdiscip. Rev. RNA.

[26] Chen C.Y., Gherzi R., Ong S.E., Chan E.L., Raijmakers R., Pruijn G.J., Stoecklin G., Moroni C., Mann M., Karin M. (2001). AU binding proteins recruit the exosome to degrade ARE-containing mRNAs. Cell.

[27] Aarabi F., Hubberten H.-M., Heyneke E., Watanabe M., Hoefgen R., De Kok L.J., Hawkesford M.J., Rennenberg H., Saito K., Schnug E. (2015). OAS Cluster Genes: A Tightly Co-regulated Network. Molecular Physiology and Ecophysiology of Sulfur.

[28] Moreno A.B., Martinez de Alba A.E., Bardou F., Crespi M.D., Vaucheret H., Maizel A., Mallory A.C. (2013). Cytoplasmic and nuclear quality control and turnover of single-stranded RNA modulate post-transcriptional gene silencing in plants. Nucleic Acids Res..

[29] Chiba Y., Johnson M.A., Lidder P., Vogel J.T., van Erp H., Green P.J. (2004). AtPARN is an essential poly(A) ribonuclease in Arabidopsis. Gene.

[30] Barreau C., Paillard L., Osborne H.B. (2005). AU-rich elements and associated factors: Are there unifying principles?. Nucleic Acids Res..

[31] Forieri I., Sticht C., Reichelt M., Gretz N., Hawkesford M.J., Malagoli M., Wirtz M., Hell R. (2017). System analysis of metabolism and the transcriptome in Arabidopsis thaliana roots reveals differential co-regulation upon iron, sulfur and potassium deficiency. Plant Cell Environ..

[32] Forieri I., Wirtz M., Hell R. (2013). Toward new perspectives on the interaction of iron and sulfur metabolism in plants. Front. Plant Sci..

[33] Shen J., Song Z., Sun Z. (2007). GO molecular function coding based protein subcellular localization prediction. Chin. Sci. Bull..

[34] Nguyen A.H., Matsui A., Tanaka M., Mizunashi K., Nakaminami K., Hayashi M., Iida K., Toyoda T., Nguyen D.V., Seki M. (2015). Loss of Arabidopsis 5’-3’ exoribonuclease AtXRN4 function enhances heat stress tolerance of plants subjected to severe heat stress. Plant Cell Physiol..

[35] Rymarquis L.A., Souret F.F., Green P.J. (2011). Evidence that XRN4, an Arabidopsis homolog of exoribonuclease XRN1, preferentially impacts transcripts with certain sequences or in particular functional categories. RNA.

[36] Von Roretz C., Di Marco S., Mazroui R., Gallouzi I.E. (2011). Turnover of AU-rich-containing mRNAs during stress: A matter of survival. Wiley Interdisc. Rev. RNA.

[37] Small I., Peeters N., Legeai F., Lurin C. (2004). Predotar: A tool for rapidly screening proteomes for N-terminal targeting sequences. Proteomics.

[38] Bannai H., Tamada Y., Maruyama O., Nakai K., Miyano S. (2002). Extensive feature detection of N-terminal protein sorting signals. Bioinformatics.

[39] Emanuelsson O., Nielsen H., Brunak S., von Heijne G. (2000). Predicting subcellular localization of proteins based on their N-terminal amino acid sequence. J. Mol. Biol..

[40] Hubstenberger A., Courel M., Benard M., Souquere S., Ernoult-Lange M., Chouaib R., Yi Z., Morlot J.B., Munier A., Fradet M. (2017). P-body purification reveals the condensation of repressed mRNA regulons. Mol. Cell.

[41] Takeo K., Ito T. (2017). Subcellular localization of VIP1 is regulated by phosphorylation and 14-3-3 proteins. FEBS Lett..

[42] Czechowski T., Stitt M., Altmann T., Udvardi M.K., Scheible W.-R. (2005). Genome-wide identification and testing of superior reference genes for transcript normalization in Arabidopsis. Plant Physiol..

[43] Zhu J., Zhang L., Li W., Han S., Yang W., Qi L. (2013). Reference gene selection for quantitative real-time PCR normalization in *Caragana intermedia* under different abiotic stress conditions. PLoS ONE.

[44] Livak K.J., Schmittgen T.D. (2001). Analysis of relative gene expression data using real-time quantitative PCR and the 2(-∆∆C(T)) Method. Methods.

[45] Schindelin J., Arganda-Carreras I., Frise E., Kaynig V., Longair M., Pietzsch T., Preibisch S., Rueden C., Saalfeld S., Schmid B. (2012). Fiji: An open-source platform for biological-image analysis. Nat. Methods.

[46] Briesemeister S., Blum T., Brady S., Lam Y., Kohlbacher O., Shatkay H. (2009). SherLoc2: A high-accuracy hybrid method for predicting subcellular localization of proteins. J. Proteome Res..

[47] Pierleoni A., Martelli P.L., Fariselli P., Casadio R. (2006). BaCelLo: A balanced subcellular localization predictor. Bioinformatics.

[48] Horton P., Park K.J., Obayashi T., Fujita N., Harada H., Adams-Collier C.J., Nakai K. (2007). WoLF PSORT: Protein localization predictor. Nucleic Acids Res..

[49] Briesemeister S., Rahnenführer J., Kohlbacher O. (2010). Yloc—An interpretable web server for predicting subcellular localization. Nucleic Acids Res..

