# Supplementary material for: The Recovery from Sulfur Starvation Is Independent from the mRNA Degradation Initiation Enzyme PARN in Arabidopsis
Source: Plants (Basel). 2019 Sep 27;8(10):380. doi: 10.3390/plants8100380 (PMC6843384; doi:10.3390/plants8100380)
Supplement: Supplementary file 1 [file plants-08-00380-s001.pdf]

Supplement

Figure S1: Wildtype seedlings stained with DAPI and MitoTracker display no signal in the GFP channel

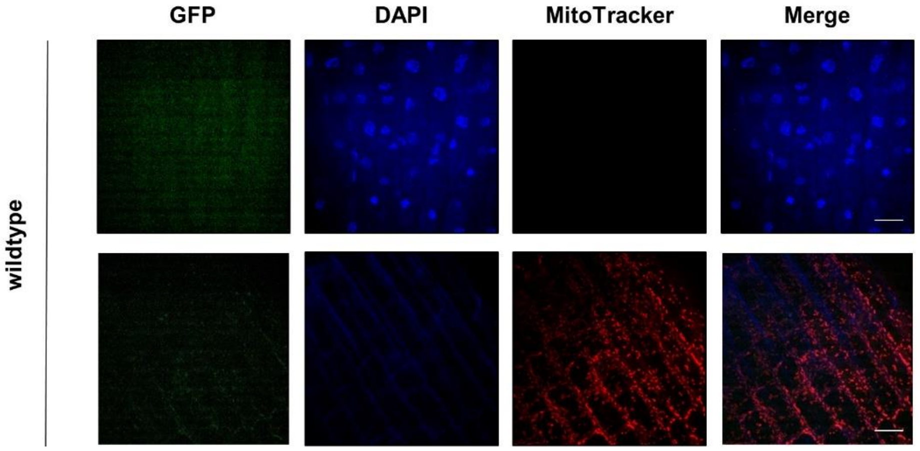

Figure S1: Roots of wildtype seedlings do not display fluorescence in the GFP signal when stained with DAPI or MitoTracker. Roots of ten-day-old seedlings grown on ½ Hoagland plates were incubated with DAPI (first row; tip section) and MitoTracker Orange (second row; elongation zone). Each column represents a different channel (GFP, DAPI, MitoTracker). The last column shows a merge of all channels. Pictures are the result of maximum intensity z-projections of slices in a z-stack. After substraction of the background, a ratio between the fluorescence signal in the GFP and the MitoTracker channel was calculated. While in the wildtype sample the GFP:MitoTracker ratio amounted to 0.09, a more than 3-fold higher ratio of 0.26 – 0.79 was measured for the AHG2-GFP samples indicating true GFP fluorescence and minimal bleed-through of the MitoTracker signal to the GFP channel. Scale bar 10 µm.

Figure S2: In roots of stressed seedlings, PARN-GFP can localize to the nucleus in rare cases

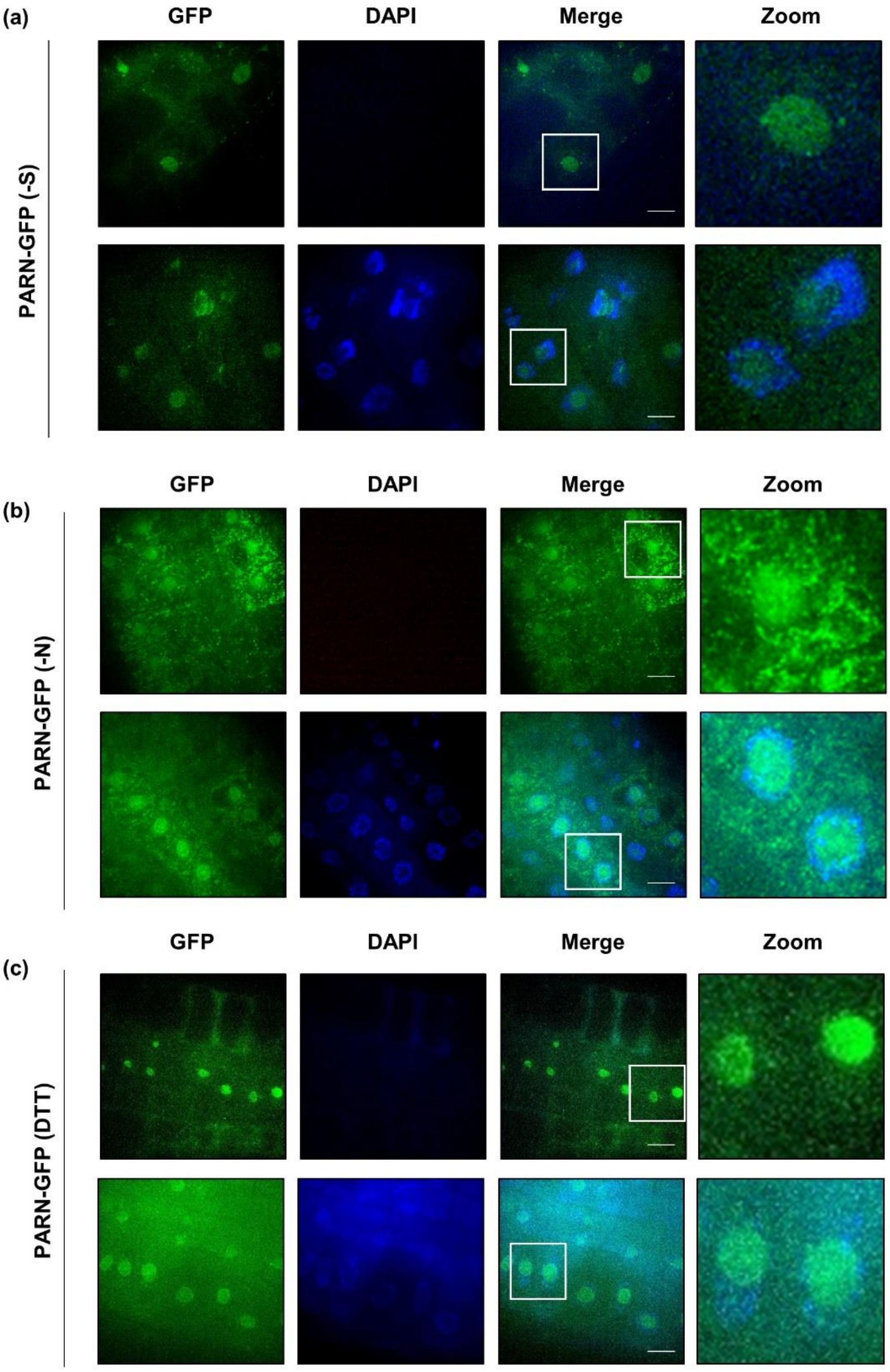

Figure S2: PARN-GFP can localize to the nucleus upon sulfur and nitrogen starvation as well as reductive stress. For (a) and (b), PARN-GFP seedlings were grown for ten days on ½ Hoagland plates devoid of sulfur or nitrogen (0 µM) respectively. To induce reductive stress (c), seedlings grown under full nutrient supply were floated in 10 mM DTT in ½ Hoagland liquid medium for 30 min. The first row shows roots imaged without any dye, whereas the second row shows samples which were incubated with 2 mg ml^{-1} DAPI. The first and second column provide images taken in the GFP and the DAPI channel whereas the third column shows a merge of the two channels. The last column represents a zoom of the merge. Each picture is the result of a maximum intensity z-projection of several images representing slices in a z-stack. Scale bar 10 µm.

Figure S3: AGS1-GFP does not change its subcellular localization upon nutrient starvation.

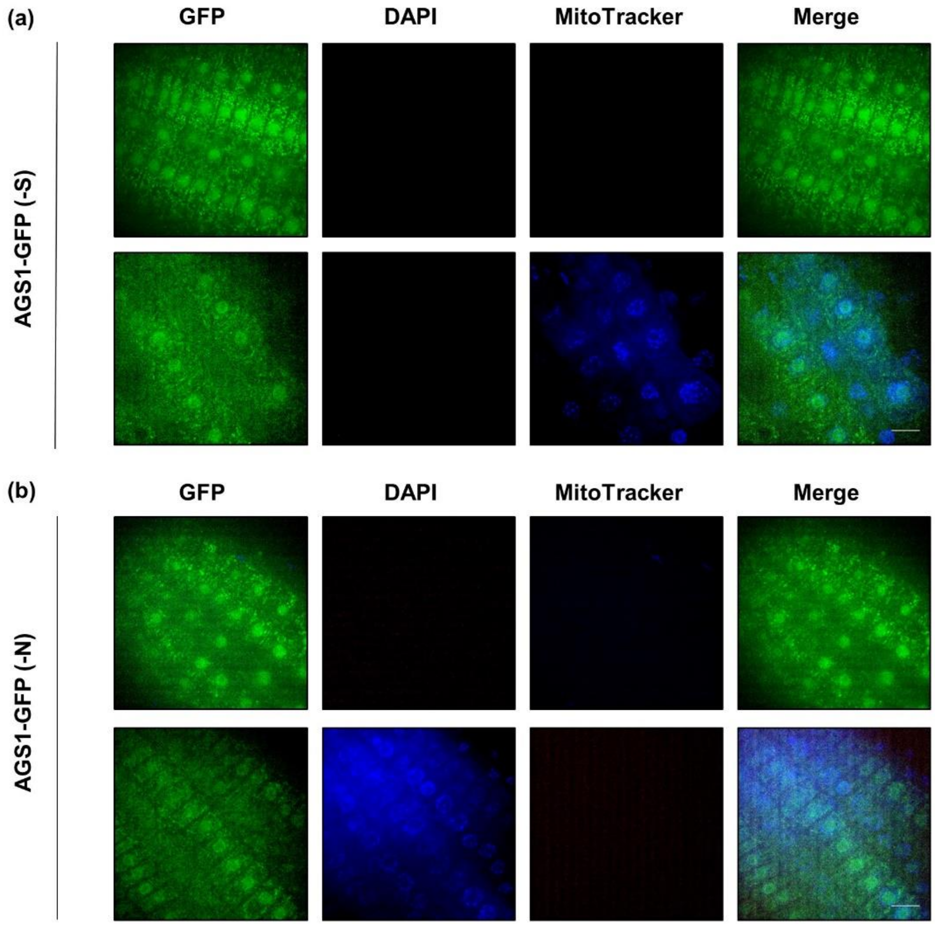

Figure S3: AGS1-GFP does not change its subcellular localization upon sulfur (a) or nitrogen (b) starvation, but remains in the nucleus. Roots of ten-day-old AGS1-GFP seedlings grown on ½ Hoagland plates depleted of sulfur or nitrogen (0 μM each) were left untreated (first row) or incubated with DAPI (second row). Each column represents a different channel (GFP, DAPI, MitoTracker). The last column shows a merge of all channels. Pictures are the result of maximum intensity z-projections of slices in a z-stack. Scale bar 10 μm.
